# Abnormal Resting-State Network Presence in Females with Overactive Bladder

**DOI:** 10.3390/biomedicines11061640

**Published:** 2023-06-05

**Authors:** Ulrich Mehnert, Matthias Walter, Lorenz Leitner, Thomas M. Kessler, Patrick Freund, Martina D. Liechti, Lars Michels

**Affiliations:** 1 Department of Neuro-Urology, Balgrist University Hospital, University of Zürich, 8008 Zürich, Switzerland; 2 Department of Urology, University Hospital Basel, University of Basel, 4031 Basel, Switzerland; 3 Spinal Cord Injury Center, Balgrist University Hospital, University of Zürich, 8008 Zürich, Switzerland; 4 Department of Neurophysics, Max Planck Institute for Human Cognitive and Brain Sciences, 04103 Leipzig, Germany; 5 Wellcome Trust Centre for Neuroimaging, UCL Institute of Neurology, London WC1N 3AR, UK; 6 Neuroscience Center Zürich, University of Zürich and Swiss Federal Institute of Technology Zürich, 8057 Zürich, Switzerland; 7 Department of Neuroradiology, University Hospital Zürich, University of Zürich, 8091 Zürich, Switzerland; 8 Clinical Neuroscience Center, University Hospital Zürich, 8091 Zürich, Switzerland

**Keywords:** resting-state magnetic resonance imaging, functional connectivity, overactive bladder, supraspinal control, urinary urgency, urgency urinary incontinence, neuro-urology, early diagnosis research

## Abstract

Overactive bladder (OAB) is a global problem reducing the quality of life of patients and increasing the costs of any healthcare system. The etiology of OAB is understudied but likely involves supraspinal network alterations. Here, we characterized supraspinal resting-state functional connectivity in 12 OAB patients and 12 healthy controls (HC) who were younger than 60 years. Independent component analysis showed that OAB patients had a weaker presence of the salience (Cohen’s d = 0.9) and default mode network (Cohen’s d = 1.1) and weaker directed connectivity between the fronto-parietal network and salience network with a longer lag time compared to HC. A region of interest analysis demonstrated weaker connectivity in OAB compared to HC (Cohen’s d > 1.6 or < −1.6), particularly within the frontal and prefrontal cortices. In addition, weaker seed (insula, ventrolateral prefrontal cortex) to voxel (anterior cingulate cortex, frontal gyrus, superior parietal lobe, cerebellum) connectivity was found in OAB compared to HC (Cohen’s d > 1.9). The degree of deviation in supraspinal connectivity in OAB patients (relative to HC) appears to be an indicator of the severity of the lower urinary tract symptoms and an indication that such symptoms are directly related to functional supraspinal alterations. Thus, future OAB therapy options should also consider supraspinal targets, while neuroimaging techniques should be given more consideration in the quest for better phenotyping of OAB.

## 1. Introduction

Overactive bladder (OAB) is a syndrome or symptom complex with the main lower urinary tract symptom (LUTS) urinary urgency, which is usually associated with urinary frequency and nocturia, with or without urgency urinary incontinence [1]. It affects millions of people worldwide and substantially impairs their quality of life, particularly if it is associated with urinary incontinence [2,3]. In addition, OAB places a substantial economic burden on any healthcare system [4,5,6,7].

The diagnosis of OAB is currently still based exclusively on anamnestic symptoms, whereby undefined “obvious” pathologies should be ruled out beforehand [1,8]. As a result, the diagnostic approach remains very superficial and does not allow any knowledge or statement about the cause of the OAB symptoms [9]. However, due to the lack of understanding about the underlying causes of OAB, targeted therapy is difficult to achieve [10], leading to the need to try a variety of therapies that are often unsuccessful in the long term [11,12,13].

Given the nature of OAB symptoms and the complex neuronal control of the lower urinary tract (LUT), it is reasonable to assume that changes in this neuronal control are of relevance in the pathophysiology of OAB. The brain, in particular, plays a crucial role in controlling the LUT, as it learns to do so during infancy and contains the most relevant LUT control areas [14,15]. However, natural aging processes and physical or psychological injuries and illnesses can cause subtle structural and functional changes in the brain, which may impair its ability to control the LUT. These alterations may be neurologically asymptomatic and difficult to detect with conventional clinical tools but can lead to symptomatic impairments of LUT control and function. Previous neuroimaging studies have supported this consideration [16,17], which may also explain why OAB prevalence increases with age, similar to the increase in cognitive impairments with age [3,17]. Although neuroimaging studies have provided some insights into the areas involved in supraspinal control of the LUT, our understanding of the functional connections between these brain areas and their potential alterations in OAB remains limited. Specifically, it is unclear whether younger OAB patients (<60 years) without cognitive impairment exhibit changes in supraspinal functional connectivity (FC) associated with OAB symptoms in the absence of any other apparent pathology.

Resting-state functional magnetic resonance imaging (rs-fMRI) can help to identify alterations in central resting-state networks, such as the default mode network (DMN), frontoparietal network (FPN), and the salience network (SAN) [18,19]. These large-scale brain networks are involved in a variety of cognitive and motor functions, including attention, memory, decision-making, and motor control [19]. The SAN is of particular interest in relation to LUT control because it is responsible for integrating information from both the internal (i.e., level of bladder filling) and external environment (i.e., availability of a location to empty the bladder) and facilitating behavioral responses (i.e., postpone micturition). Furthermore, the SAN is an important regulator and mediator between other large-scale brain networks, particularly the DMN and the FPN, contributing to complex mental processes such as detection and filtering or amplification of ascending signals, evaluation of information, and initiation of appropriate actions, allowing for attentional control [20]. With its core region, the anterior insular cortex (AIC), the SAN is also involved in perceiving and responding to homeostatic demands and emotional awareness and is considered a “detector” of the interoceptive state (i.e., altered levels of bladder filling) [21,22,23,24,25]. Hence, The SAN is involved in several processes relevant to controlling the LUT.

The question under investigation in this exploratory rs-fMRI study is whether alterations in supraspinal connectivity, including the presence and interaction of central resting-state networks, such as the SAN and DMN, but also region of interest (ROI)-to-ROI and seed-to-voxel interactions are already present and measurable using rs-fMRI in female OAB patients (<60 years) without bladder task/stimulation compared to healthy controls (HC) and if such alterations in FC at least partly correlate with their OAB symptoms. Considering the aforementioned relevance of supraspinal processes in controlling the LUT, their potential role in the etiology of OAB, previous fMRI findings [26,27,28], and the capabilities of rs-fMRI, our biological hypothesis is that OAB patients present weaker supraspinal connectivity patterns compared to HC that can be measured and visualized with rs-fMRI. Based on our prior fMRI study [28] that showed lower activity in the AIC and ventrolateral prefrontal cortex (VLPFC) in OAB patients compared to HC during bladder filling and draining, we hypothesized that the SAN would exhibit weaker presence and FC in the OAB group compared to HC and that FC patterns related to the AIC and VLPFC would be reduced in OAB patients. We further hypothesized that these latter alterations in FC will correlate with the symptoms of the OAB patients as a sign of their reduced supraspinal LUT control.

## 2. Materials and Methods

### 2.1. Participants

We enrolled right-handed females (age 18–55 years) who were suitable for magnetic resonance (MR) imaging and were either diagnosed with OAB or were healthy controls (HCs) without LUTS. Table 1 provides an overview of the inclusion and exclusion criteria. To assess eligibility, each participant underwent clinical assessments that included a medical history review, neuro-urological examination, completion of a 3-day bladder diary, urinalysis, and urodynamic investigation.

### 2.2. Questionnaires

In order to assess the absence or degree/severity of LUTS, anxiety, depression, and cognitive impairment, all participants were asked to complete validated German versions of a battery of standardized questionnaires. The International Consultation on Incontinence Modular Questionnaire (ICIQ, Bristol Urological Institute, Southmead Hospital, Bristol, UK) on LUTS for women (ICIQ-FLUTS) is a validated 12-item questionnaire consisting of three subscales (filling, voiding, and incontinence) that has been widely used to measure lower urinary tract symptoms in women [29]. The Overactive Bladder Questionnaire short form (OAB-q SF, Pfizer patient-reported outcomes, Pfizer, New York, NY, USA) is a validated questionnaire that assesses the patient perception of symptom bother and impact on health-related quality of life (QoL) [30]. It comprises 6 items that measure symptom bother (lowest and highest possible raw scores are 6 and 36; possible raw score range is 30) and 13 items that measure health-related QoL (lowest and highest possible raw scores are 13 and 78; possible raw score range is 65). Raw scores are then transformed according to Coyne et al. [30], i.e., for symptom severity: [(actual raw score − lowest possible raw score)/possible raw score range] × 100, resulting in a range from 0 (=best possible) to 100 (=worst possible): and health-related QoL: [(highest possible raw score − actual raw score)/possible raw score range] × 100, resulting in a range from 0 (=worst possible) to 100 (=best possible). The Hospital Anxiety and Depression Scale (HADS) is a validated questionnaire consisting of two subscales, i.e., anxiety and depression, with 7 items each, and was found to be a reliable instrument for detecting states of depression and anxiety [31]. The Mini-Mental State Examination (MMSE) is a 30-point questionnaire that is used in clinical and research settings to screen for cognitive impairment [32].

### 2.3. Pre- and Post-Measurement Procedures

Prior to undergoing magnetic resonance imaging (MRI), all participants were required to refrain from consuming any products containing caffeine or nicotine for at least six hours. Upon arrival, participants emptied their urinary bladder, underwent urinalysis to rule out urinary tract infection (UTI) or pregnancy, and changed into standard scrubs with all ferromagnetic items removed. In order to monitor for adverse events such as UTI, participants were interviewed via telephone or email within one week after the MRI session.

### 2.4. MRI Recording

We collected structural and functional MRI (fMRI) data using a Philips Ingenia 3-Tesla scanner (Philips Medical Systems, Best, the Netherlands) equipped with a 15-channel Philips Sense head coil. The acquisition included high-resolution three-dimensional T1-weighted turbo field gradient echo pulse sequences that covered the entire cerebrum and cervical spinal cord (5:05 min, isotropic resolution: 1 mm^3^, field of view: 256 × 256 × 180 mm, matrix: 256 × 256 mm, slices: 180 slices, scan time: 305 s, repetition time (TR) = 6.92 ms, echo time (TE) = 3.1 ms, no inter-slice gap, and flip angle = 8°).

The rs-fMRI time series were acquired with a field echo (i.e., gradient echo) planar imaging pulse sequence. Thirty-four axial slices covered the entire cerebrum in ascending order (TR = 2000 ms, TE = 16 ms, slice thickness = 3 mm, inter-slice gap = 1 mm, flip angle = 80°, field of view = 240 × 135 × 240 mm, and reconstructed image matrix 80 × 80 voxels, voxel resolution: 3 × 3 mm, scan duration: 410 s). All images were obtained in an oblique axial orientation covering the entire brain, including the cerebellum and rostral brainstem (including the pons). The first four scans were dummy scans to reach steady-state magnetization. We examined rs-fMRI during an empty bladder condition (subjects fixated on a central cross during scanning).

### 2.5. Resting-State fMRI Connectivity Analysis

We used the CONN toolbox (version 20a, https://web.conn-toolbox.org/ (accessed on 12 September 2022)) [33] implemented in MATLAB R2021a (MathWorks, Natick, MA, USA) and SPM12 (https://www.fil.ion.ucl.ac.uk/spm/software/spm12/ (accessed on 12 September 2022)) to perform fMRI data pre-processing and rs-fMRI connectivity analyses. CONN employs an anatomical component-based noise correction method (CompCor), which enhances specificity and accuracy, and enables greater consistency across scans [34]. Additionally, CONN includes nuisance regressors that address outlier data points and movement time courses. In order to reduce noise and confounding signals not related to neuronal activity associated with FC, the six motion parameters, white matter, and cerebrospinal fluid were included as regressors of no interest [35]. To further remove potential confounding effects on the fMRI signal by head motion, we applied scrubbing (variable number of noise components (one for each identified outlier scan during the outlier identification pre-processing step)). Additional pre-processing steps included denoising, MNI normalization to the MNI template brain (to allow group-level analysis), spatial smoothing (Gaussian kernel with six mm FWHM), and band-pass filtering (0.01–0.1 Hz). The latter was used to remove linear drift artifacts and high-frequency noise. FC distributions can show extremely large inter-subject variability and skewed distributions with varying degrees of positive biases, consistent with the influence of global or large-scale physiological and subject-motion effects. After the denoising step, FC distributions showed approximately centered (normal) distributions, with small but noticeably larger tails on the positive side and considerably reduced inter-subject variability. We checked the quality of the denoising procedure outputs by computing Quality Control-FC correlations [36]. Specifically, a QC-FC correlation value was computed for each randomly selected pair of points within the brain, and then we displayed the distribution of the resulting QC-FC correlation values. This distribution was then compared to an associated null-hypothesis distribution, with an associated percent match value quantifying the degree of similarity between the two (95% or higher match with null-hypothesis indicates lack of noticeable QC-FC associations). Thus, parametric analyses were performed for any FC group comparison (see below).

To assess the FC pattern in OAB vs. HC, we applied four FC approaches: independent component analysis (ICA), ROI-to-ROI connectivity, seed-to-voxel connectivity, and directed connectivity:

**ICA:** Twenty independent components (ICs) were extracted (default setting in CONN) for the rs-fMRI run across all subjects. ICs related to head motion or artifacts were not considered (ICs = 6). The remaining 14 ICs were labeled as follows: cerebellum, fronto-parietal attention network (3 ICs: left, right, bilateral), DMN (3 ICs: anterior, posterior, and bilateral), early and higher visual cortex, somatosensory network, auditory cortex (2 ICs), SAN (2 ICs: bilateral and supplementary motor area (SMA) only). Based on our hypothesis and to reduce the number of statistical comparisons, we focussed on three networks: bilateral DMN (1 IC), bilateral frontoparietal network (FPN, 1 IC), and bilateral SAN (1 IC).

**ROI-to-ROI connectivity:** Connectivity was estimated from 146 ROIs based on the AAL atlas [37] and two distinct ROIs from our previous paper [28], i.e., the right AIC and VLPFC. Here, we used the MNI peak coordinate with a 6 mm sphere for ROI creation.

**Seed-to-voxel connectivity:** We used two seed regions based on our previous publication [28]: (a) right AIC and (b) right VLPFC to examine its connectivity to the whole brain (voxel level) on the fine-scale functional level.

**Directed connectivity:** The MATLAB GIFT toolbox (https://www.nitrc.org/projects/gift (accessed on 12 September 2022)) and Functional Network Connectivity Toolbox (FNCT, https://trendscenter.org/software/fnc/ (accessed on 12 September 2022)) were used to estimate the temporal dynamics of FC between the DMN, FPN, and SAN (deduced from the ICA) [38]. In particular, we studied the temporal lag (in seconds, +/− 3 s) of the fMRI signal between a given network and the two other networks. This approach has been applied in other studies, e.g., comparing HC and patients with schizophrenia or cocaine-dependent participants [39,40]. In the first step, correlations between examined networks were estimated, and in the second step, time lags for group differences were derived. The provided lag *p*-value is calculated as part of a permutation test and represents the proportion of permuted datasets in which the correlation between three networks at a given time lag is equal to or greater than the correlation in the original data (i.e., a sign for the strength of directed connectivity). A lag *p*-value of less than 1 indicates that the observed correlation at the given time lag is higher than what would be expected by chance based on the corresponding permuted data. This suggests that the correlation is unlikely to have occurred by chance [41].

All analyses were calculated using two-tailed independent t-tests. All results are reported at *p* < 0.05 corrected, i.e., cluster-corrected [42] or corrected using the False Discovery Rate (FDR) correction [43]. For the ROI-to-ROI and seed-to-voxel FC, we computed Cohen’s d effect sizes according to the following formula:

Cohen’s d = 2 t/√(df), with n1 (HC = 12) = n2 (OAB = 12) and degree of freedom (df) = 22 [44,45,46].

In addition, to assess the effect of the main clinical symptoms, i.e., urinary urgency, urinary frequency, and urgency urinary incontinence (UUI) episodes, we included the data from the 3-day bladder diaries on all three symptoms as covariates of interest into the statistical model and performed seed (right AIC and right VLPFC) to voxel Pearson correlations (converted to t-values).

### 2.6. Statistical Analyses of Population Characteristics (Including Clinical and Behavioral Data)

Descriptive statistics of population characteristics are displayed as medians and range from the lower (25th percentile) to upper (75th percentile) quartile for all continuous and discrete variables (Table 2), under the assumption that not all variables will follow a normal distribution [47,48] and considering the exploratory nature of this study with a rather small sample size. Group differences were calculated by subtracting the median value of the healthy control population from the median value of the OAB group, and 95% confidence intervals (CIs) were obtained by bootstrapping (1000 iterations). The dichotomous variable, i.e., the presence of detrusor overactivity, is displayed as a number and proportion; 95% confidence intervals were derived from a two-sample test of proportions. Statistical analysis was performed in Stata (version 17, StataCorp LLC, College Station, TX, USA).

## 3. Results

Twenty-four right-handed female participants, 12 with OAB (40 years, 31–43) and 12 HCs without LUTS (34 years, 28–45), were recruited and included in the analyses. According to their selection (Table 1), OAB and HC groups showed characteristic differences for 3-day bladder diary parameters, ICIQ-FLUTS scores (except voiding subscale), OAB-q SF scores, filling cystometry parameters (i.e., strong desire to void, maximum cystometric capacity, and presence of detrusor overactivity), and voided volume of the pressure flow study (Table 2). Neuro-urological examination (including tests of urogenital sensation, bulbocavernosus reflex, anocutaneous reflex, anal sphincter tone, and voluntary anal sphincter contraction) revealed normal findings in all participants. There were no adverse events during the baseline assessment, MRI session, or follow-up.

### 3.1. Independent Component Analysis

**Figure 1:**
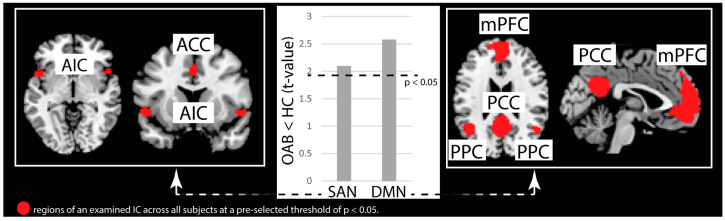
Summary of findings for the independent component (IC) analysis. The panel on the left illustrates the salience network (SAN) derived across all subjects at a pre-selected threshold of *p* < 0.05, with major brain regions (red spots) such as the anterior insular cortex (AIC) and the anterior cingulate cortex (ACC). The panel on the right illustrates the default mode network (DMN) derived across all subjects at a pre-selected threshold of *p* < 0.05, with major brain regions (red spots) such as the posterior cingulate cortex (PCC), medial prefrontal cortex (mPFC), and posterior parietal cortex (PPC). The panel in the middle shows a bar chart of the statistical differences (t-value) of the group contrast healthy control (HC) vs. overactive bladder (OAB) in the described ICs, i.e., SAN (t = 2.1, Cohen’s d = 0.9) and DMN (t = 2.58, Cohen’s d = 1.1). Values above the dashed line (t = 1.96) indicate HC > OAB for the examined ICs with a threshold of *p* < 0.05.

The results of the ICA showed a weaker presence of the SAN and DMN in OAB patients compared to HC, as depicted in Figure 1. No group differences were observed in the other examined ICs, including FPN.

### 3.2. ROI-to-ROI Connectivity

**Figure 2:**
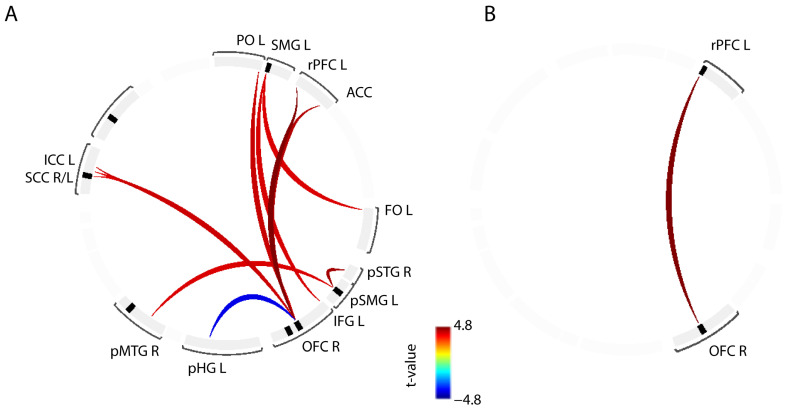
Illustration of findings from the region of interest (ROI) analysis. Connections in red depict region-to-region connectivity that is weaker in OAB compared to HC. The connection in blue depicts region-to-region connectivity that is stronger in OAB compared to HC: (**A**) Findings at *p* (voxel-threshold) < 0.001 with cluster level correction at ROI level (*p* < 0.05). (**B**) Findings at *p* (voxel-threshold) < 0.0001 with cluster level correction at ROI level. ACC (anterior cingulate cortex), FO L (frontal operculum, left), ICC L (intracalcarine sulcus, left), IFG L (inferior frontal gyrus, left), OFC R (orbitofrontal cortex, right), pHG L (parahippocampal gyrus, left), pMTG R (posterior middle temporal gyrus, right), PO L (parietal operculum, left), pSMG L (posterior supramarginal gyrus, left), pSTG R (posterior superior temporal gyrus, right), rPFC L (rostral prefrontal cortex, left), SCC R/L (supracalcarine cortex, right and left), SMG L (supramarginal gyrus, left).

The ROI-to-ROI connectivity analysis revealed that, across all examined regions, OAB patients exhibited weaker FC between various brain regions predominantly located in the prefrontal cortex, such as the inferior frontal gyrus (IFG), frontal operculum (FO), ACC, orbitofrontal cortex (OFC), and rostral prefrontal cortex (rPFC) (Figure 2A, Table 3). Only one connection, specifically the right OFC—left parahippocampal gyrus (pHG) was found to be stronger in OAB patients compared to HC.

At a more conservative statistical threshold (Figure 2B, *p-voxel* < 0.0001 with *p* < 0.05 cluster level correction), the connection between the right OFC and left rPFC remained weaker in the OAB group.

### 3.3. Seed-to-Voxel Connectivity

**Figure 3:**
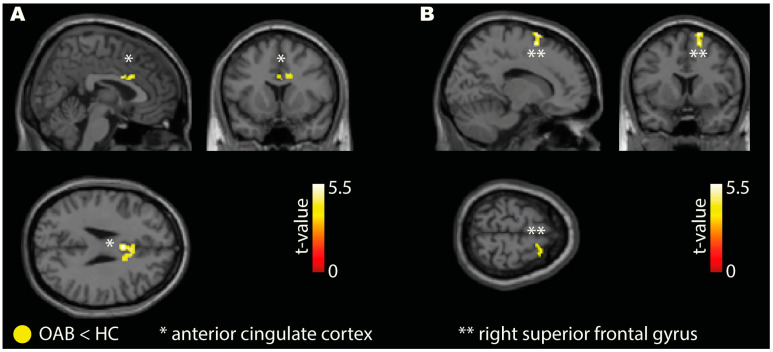
Exemplary visualization of the results from the seed-to-voxel analysis: (**A**) Overactive bladder (OAB) patients showed weaker connectivity compared to healthy controls (HC) from the seed (right anterior insular cortex) to the anterior cingulate cortex. (**B**) OAB patients showed weaker connectivity compared to HC from the seed (right VLPFC) to the right superior frontal gyrus. Results are shown at *p* (voxel-threshold) < 0.001 with cluster level correction at region of interest level (*p* < 0.05).

Our findings revealed weaker FC in the OAB group compared to HC for both seeds (right AIC and right VLPFC). The right AIC seed demonstrated weaker connectivity in the OAB group with the ACC (Figure 3A), IFG, SPL, MFG, calcarine cortex, and cerebellum (Table 4). The right VLPFC seed showed weak connectivity to the right superior frontal gyrus in the OAB group (Figure 3B, Table 4). Additional analysis for the periaqueductal gray as seed region is reported in the Supplementary Materials.

### 3.4. Directed Connectivity

**Figure 4:**
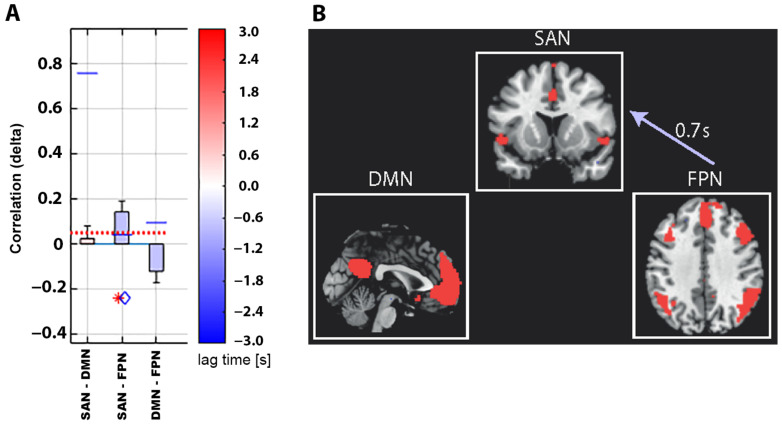
(**A**) Box plot chart of the group differences in directed functional connectivity between the three large-scale brain networks: the salience network (SAN), the default mode network (DMN), and the frontoparietal network (FPN). Positive correlations (*y*-axis) indicate overactive bladder (OAB) patients < healthy controls (HC). The SAN showed weaker connectivity (red asterisk indicating *p* < 0.05) to the FPN comparing OAB patients to HC. The blue lines indicate correlation *p*-values ((SAN-DMN): *p* = 0.76, SAN-FPN: *p* = 0.04, DMN-FPN: *p* = 0.10). The red dotted line indicates the *p*-value threshold of *p* < 0.05 corrected using False Discovery Rate (FDR). The lag time group difference (blue diamond, lag *p*-value = 0.41) SAN-FPN was −0.7 s, i.e., the SAN is activated 0.7 s later than the FPN in OAB patients compared to HC. (**B**) Graphical network illustration of temporal group difference in directed functional connectivity. The lag time (FPN to SAN) was 0.7 s, i.e., the SAN was activated 0.7 s later, comparing OAB patients to HC. Results are shown at *p* < 0.05 (corrected using the False Discovery Rate (FDR)).

The directed connectivity analysis revealed that the SAN-FPN connectivity was weaker in the OAB group. In addition, the SAN was activated on average 0.7 s later than the FPN in OAB patients compared to HC (Figure 4).

### 3.5. Relation of OAB Symptoms to Functional Connectivity

Correlating the number of urinary urgencies, urinary frequency, and UUI episodes from the 3-day bladder diary with the seed (right AIC and right VLPFC) to voxel connectivity in the OAB group revealed positive (=increasing with the number of urinary urgency/frequency or UUI episodes) and negative (=decreasing with the number of urinary urgency/frequency or UUI episodes) correlations summarized in Table 5a–c.

## 4. Discussion

This study supports our biological hypothesis that patients with OAB exhibit reduced resting-state brain FC compared to healthy controls (HCs). Specifically, we observed weaker FC in components of the SAN as well as the frontal and prefrontal cortices among patients with OAB.

In the context of LUT control, an intact SAN connectivity and interaction with the DMN and FPN would refer to the ability to timely detect ascending bladder signals, i.e., desire to void, to appropriately evaluate this information, i.e., how and to what extent has the desire to void increased over time and how strong is the desire to void in relation to the spatial and temporal possibility of going to the toilet, and to take according action, i.e., going to the toilette immediately or postpone it to a later more convenient time point. In patients with OAB, these abilities seem to be lost or at least impaired, represented by the reduced SAN, ROI-to-ROI (i.e., ACC to OFC, rPFC to OFC), and seed (AIC, VLPFC) to voxel (i.e., ACC and right superior frontal gyrus, SFG) connectivity.

In line with our previous findings using task-based fMRI [28], demonstrating lower AIC and VLPFC activity in response to bladder-related sensations in OAB compared to HCs, we now also see FC deficits in OAB compared to HCs in brain networks strongly involving insular and PFC activity. In addition, by using directed connectivity analysis, we could show that in OAB, the temporal interplay between those networks, i.e., FPN to SAN, is reduced compared to HC. The ability to temporally orchestrate communication between functional brain networks, however, is essential to prepare for and initiate goal-oriented action and strategy [20]. Hence, our current observation that large-scale brain networks such as SAN and DMN are less strongly represented in OAB patients and that switching between these networks, i.e., FPN to SAN, occurs more incompletely and less rapidly compared to HC, supports and further extends the hypothesis from our previous study that OAB is associated with altered sensory processing and attentional control at the supraspinal level.

Previous neuroimaging studies in humans have demonstrated the importance of the dorsal ACC/medial SFG and the bilateral AIC/FO brain regions during task control, involving task initiation, maintenance, and feedback/adjustment [49,50]. Furthermore, there is evidence that the dorsal ACC and medial SFG actively exerts top-down control over sensory and limbic brain regions [49,51,52]. Reduced activity and connectivity among these regions and further frontal areas, such as the PFC and OFC, may explain detrusor overactivity (DO) and UUI because of insufficient inhibitory/top-down control. Both DO and UUI are frequently associated with OAB and are highly prevalent in the currently investigated OAB group (58% demonstrated DO during urodynamics, and 66% presented with UUI in the 3-day bladder diary).

While comparability with previous MRI studies investigating differences in resting-state FC between HC and individuals with OAB/UUI is often challenging and therefore needs to be viewed with some caution due to differences in population characteristics, imaging parameters, and statistical methods, our current results appear to be in general agreement with the few available similar studies [26,53,54,55,56]: OAB/UUI patients demonstrate weaker FC compared to HC among areas relevant for LUT control and in regard to the large-scale brain networks. Nardos et al. showed that women with UUI had a lack of FC with bladder filling among brain areas involved in interoception (insula), afferent function integration (ACC), and decision-making (middle frontal gyrus) [26]. Similarly, Zuo et al. demonstrated that OAB patients had decreased FC within various networks, including the sensorimotor-related network, the dorsal attention network, the dorsal visual network, and the left FPN [54]. In another study by Zuo et al., OAB patients presented decreased short- and long-range FC strength in several brain regions, including the medial SFG, ACC, and posterior cingulate cortex [53]. Switching among large-scale brain networks such as the DMN, the SAN, and the FPN typically involves long-range FC, which is disturbed in those OAB patients and, thus, is in agreement with our findings. Moreover, Ketai et al. demonstrated, in line with our results, that controls had generally higher resting-state FC, particularly in relation to the VLPFC, compared to UUI patients [55]. Finally, Wang et al., in a most recent study, showed that the amplitude of low-frequency fluctuations (ALFF) is decreased in OAB compared to HC in most regions [56]. Particularly, the lower ALFF in the frontal cortex in OAB (left medial SFG) was negatively correlated with the OAB symptom scores [56], i.e., fewer frontal connectivity and control results in more OAB symptoms, which also corresponds to our findings (Table 5, negative correlations of PFC connectivity with OAB symptoms). The only ALFF connectivity that was higher in OAB compared to HC was in the right hippocampus [56]. Interestingly, this is quite similar to the only ROI-to-ROI connectivity that was also stronger in OAB compared to HC in our study: left orbitofrontal cortex to left parahippocampal gyrus. Although the parahippocampus and hippocampus are not identical, they are closely related anatomically and functionally. Both the parahippocampal gyrus and the hippocampus are involved in a variety of cognitive processes, including memory formation and retrieval, spatial navigation, contextual processing, and emotion regulation [57]. In the context of LUT function, the parahippocampal gyrus and the hippocampus may not be directly involved but in conjunction with the amygdala, thought to be associated with the processing of interoceptive signals related to bladder fullness, which can influence the decision to initiate or postpone urination [16]. This is supported by animal studies demonstrating that mineralocorticoid- and glucocorticoid-mediated stimulation of the amygdala resulted in urinary bladder hypersensitivity [58], whereas lesions to the amygdala caused less acute urinary bladder hypersensitivity in response to electric foot shock stress [59]. The result of an earlier PET study, which was able to show that UUI patients have reduced regional cerebral blood flow in the left amygdala in connection with improved symptoms under treatment with chronic sacral neuromodulation (>6 months), also points in a similar direction [60]. The amygdala is a crucial component in generating emotions, particularly those associated with fear, and in mediating unconscious and conscious reactions to emotional events such as the fear of leakage during moments of urgency. Therefore, the parahippocampal/amygdala circuit is believed to be particularly involved in the emotional and safety-related aspects of bladder control [15,57]. In the event of a loss of control, as with OAB/UUI, this parahippocampal/amygdala circuit could become more involved in searching for alternative solutions if the “actual” control networks can no longer be accessed to a sufficient extent. A further example of this lack of control in the OAB/UUI condition in the study by Wang et al. is the diminished FC between the mPFC and the amygdala, which usually would allow top-down control to support emotional regulation and prevent inappropriate emotional expression [56]. A diminished fronto-amygdala FC may not only make emotional regulation more difficult but also the control of subsequent processes, which can ultimately lead to an increase in the stressful urgency situation and, finally also, to incontinence.

However, there is also evidence that the FC in OAB patients is not always inferior to that in HC in all areas. Zuo et al. showed that short-range FC strength was increased in OAB in the middle frontal gyrus, right precentral gyrus, and bilateral caudate nucleus, which was interpreted as compensatory pathways related to impaired response inhibition of the brain–bladder control network [53]. Ketai et al. demonstrated greater FC between the MCC and dorsolateral prefrontal cortex (DLPFC) in UUI patients, which was interpreted as top-down support for goal-directed orientation (e.g., maintaining continence) since the DLPFC is assigned to both the dorsal FPN (attention) and lateral FPN (control) [55,61]. Wang et al. found in an ROI-to-ROI FC analysis that patients with OAB had higher FC compared to HC between the bilateral caudate nucleus (CN) and bilateral dorsal SFG, the bilateral CN and bilateral SMA, the bilateral thalamus and SMA; the left CN and bilateral medial SFG, the left CN and bilateral ACC, and the left CN and left insula [56]. The latter FC between CN and insular cortex, and CN and ACC was interpreted as an enhanced function of the interoceptive network that related to the UUI experience results in an adaptive stimulation enhancement, similar to the initial task-based fMRI findings by Griffiths et al. [62]. The stronger FC to the SMA from CN and thalamus in UUI patients may reflect in these patients the necessity to enhance the early involvement of motor areas for better pelvic floor muscle control [63,64] in conditions of urinary urgency to help prevent incontinence [56].

These aforementioned apparently discrepant results may very well represent compensatory changes in the context of OAB/UUI, as described by the respective authors themselves. As a result, they would not necessarily be contradictory, but possibly even complementary, since they remain part of a spectrum of the same problem if one assumes, for example, that there are most probably different types and stages of OAB in relation to their development and clinical expression [65,66,67]. Hence, there may be different stages, strategies, and requirements for compensation. However, neural compensation describes the ability to use flexible brain networks after pathology or normal aging disrupts those typically recruited to maintain a particular task performance [68]. Based on the study situation to date, the question arises as to whether the findings of alternate brain activity/connectivity in OAB/UUI patients really represent a compensatory mechanism helping to prevent or reduce symptoms or are rather the actual problem resulting in urinary urgency or UUI. Tadic et al. investigated the difference in supraspinal activity between OAB patients who had DO during the MRI investigation and those who did not [65]. OAB patients with DO presented with more activity in areas adjacent to the SMA and the postcentral gyrus at low and high bladder filling volumes but less activity in the frontal cortices compared to those without DO. This was interpreted as compensation for the lack of frontal inhibitory control to better activate the pelvic floor and sphincter muscles by recruiting additional motor areas, ultimately to avoid UUI despite DO. This appears plausible, but unfortunately, it was not clarified if they were successful in avoiding UUI during measurement; at least, they were not successful in daily life (3.6 ± 1.9 UUI episodes/24 h) [65]. In most previous FC studies in the context of OAB/UUI [53,54,55,56], the patient groups were often only described inadequately (no information on bladder diaries or urodynamics), so that a more precise characterization of the severity of the OAB and whether compensatory mechanisms have taken effect is not possible here either. When correlating the seed-to-voxel connectivity with the symptom severity in our OAB group, the findings indicate that FC from the AIC to areas in the occipital and temporal lobe (Table 5a), which are not typically associated with supraspinal LUT control [16,69,70], was positively associated with higher numbers of urgency episodes. This supports the theory that such atypical connections may not be compensatory but rather contribute to the problem. The results of Wang et al. point in a similar direction; when increasing OAB symptoms, the lower the ALFF in the SFG [56]. On the other hand, we also found negative correlations between the numbers of urgency episodes and FC to areas involved in sensorimotor function (pre- and postcentral gyrus) and frontal inhibitory control (IFG) [71], supporting the idea of previous studies that such areas may be indeed used as a compensatory mechanism to reduce urgency symptoms and prevent subsequent UUI [65,67]. Interestingly, we observed a positive correlation between the number of UUI episodes and FC between the VLPFC and an area in the brainstem (as shown in Table 5c) that is similar to what has been previously described as the pontine micturition center (PMC) [72]. Consistent with our findings, Griffiths et al. also reported activity in the PMC in patients with UUI [73]. Premature activation of the micturition reflex via the PMC due to a lack of or insufficient inhibitory frontal control can understandably facilitate UUI. On the other hand, and thus in agreement with the above results, there are negative correlations to UUI, i.e., less incontinence, with connections to the frontal areas (IFG) and to the precuneus (Table 5b), which is assigned to the DMN, relevant to integrate both external and self-generated information, and supposed to play a central role in the modulation of conscious processes [74].

These findings may offer an explanation to the questions surrounding the current first-line therapy for OAB, which involves antimuscarinic drugs. Specifically, they may shed light on the reasons why there is a relevant placebo effect, as well as why many patients ultimately discontinue therapy due to a poor effect-to-side-effect ratio, despite the initial therapeutic benefit [75]: The therapy for OAB may be initiated at the wrong level, i.e., at the intrinsically healthy end organ. Although this approach may lead to initial symptom reduction, it fails to address the underlying causal problem, resulting in no lasting improvement in the long term. It is, therefore, not surprising that a recent systematic review and network meta-analysis of the most relevant OAB therapies showed that sacral neuromodulation, which is thought to work by modulating not only spinal cord reflexes but also brain networks [60,76,77], demonstrated the most favorable results for reducing micturition frequency, urgency episodes, and UUI episodes [78].

Despite that, previous neuroimaging studies have indicated that altered supraspinal processes play a role in OAB [16,26], yet the focus on diagnosis and management of OAB continues to be primarily LUT-focused. Our current study contributes further evidence to the need for a reevaluation of the diagnosis and therapy of OAB beyond the LUT, which is in line with the demand for better phenotyping of the OAB syndrome to enable more targeted therapy [10]. However, to improve the phenotyping of this symptom complex, it will not be sufficient to simply subdivide the patients based on continent vs. incontinent or with-DO vs. without-DO. Instead, a comprehensive range of diagnostic tools, including medical history; diaries; symptom questionnaires; laboratory blood and urine tests; cystoscopy; urodynamics; and histopathological, neurophysiological, psychosomatic, and neuroimaging assessments will be necessary to examine and identify pathognomonic patterns that can allow for a more precise determination of an OAB phenotype.

### Limitations

The MRI investigation was only performed once; therefore, no statement can be made about the dynamics or development of the supraspinal connectivity. In this regard, longitudinal data would be relevant in the future in order to understand the course and development of the supraspinal changes in relation to the symptoms and clinical parameters. Despite all efforts to recruit a well-defined group of OAB patients for this study (i.e., strict in-/exclusion criteria according to current knowledge and standard with extensive screening, Table 1), a certain inter-subject variance with regard to the exact cause and development of OAB symptoms, which can influence our results, cannot be completely ruled out. A generalization of the results to all OAB patients is not permissible due to the mostly multifactorial etiology. The study population is too small for further subgroup analysis.

## 5. Conclusions

This rs-fMRI study demonstrates that OAB patients, compared to HCs, have deficits in resting-state brain connectivity. Particularly large-scale brain networks such as the DMN and the SAN, as well as their interactions, seem to be disturbed in OAB. Moreover, connectivity to and within frontal/prefrontal cortices is diminished in OAB compared to HCs. Such alterations correlated positively with symptom severity, i.e., the number of urinary urgency and UUI episodes. Hence, these findings provide further evidence that etiologies and, consequently, therapy targets of OAB must be considered beyond the LUT, i.e., on the supraspinal level. Especially concerning the efforts to improve the phenotyping of the OAB and to assess the effect of OAB therapies on neuronal LUT control, it will be increasingly necessary to consider and advance the use of neuroimaging methods in this area of functional urology and neuro-urology.

## Figures and Tables

**Table 1:** Inclusion and exclusion criteria for all subjects.

Group	Inclusion Criteria	Exclusion Criteria
All	▪ Right-handed ▪ Female sex ▪ Age limits: 18–55 years ▪ MR suitability ▪ Written informed consent	▪ Pregnancy or breastfeeding ▪ Any neurological, psychological, metabolic, or cardiovascular disease ▪ Any craniocerebral injury or surgery ▪ Any permanent ferromagnetic implant ▪ Any previous surgery of LUT/genitalia ▪ Any anatomical anomaly of LUT/genitalia ▪ Any LUT malignancy ▪ PVR > 150 mL ▪ UTI (recurrent or acute) ▪ Any concomitant treatment for the LUT (e.g., neuromodulation, botulinum toxin intradetrusor injections) ▪ SUI ▪ Indwelling catheters or necessity to perform ISC
OAB	▪ LUTS > 6 months (3-day bladder diary) ▪ Urinary urgency episodes ≥ 6/72 h ▪ Urinary frequency > 24/72 h *	
HC	▪ Unimpaired LUT function ▪ No LUTS (3-day bladder diary) ▪ No episode of urinary urgency ▪ Urinary frequency < 24/72 h	▪ Impaired LUT function ▪ Any LUTS (3-day bladder diary) ▪ Any episode of urinary urgency ▪ Urinary frequency > 24/72 h

HC = Healthy control, MR = Magnetic resonance, LUT = Lower urinary tract, LUTS = Lower urinary tract symptoms, OAB = Overactive bladder, PVR = Post void residual, UTI = Urinary tract infection, SUI = Stress urinary incontinence, ISC = Intermittent self-catheterization. * If the fluid intake was already deliberately reduced to <1500 mL/24 h due to the urinary urgency/frequency symptoms, a frequency of 24/72 h was exceptionally accepted.

**Table 2:** Baseline characteristics of the participants.

Baseline Characteristics	OAB Group (*n* = 12)	HC Group (*n* = 12)	Group Difference (95% CI) [OAB − HC Group]
**Demographics**			
Age (years)	40 (31–43)	34 (28–45)	6 (−6, 18)
Weight (kg)	70 (57–77)	58 (56–65)	12 (1, 23)
**3-day bladder diary**			
Fluid intake per 72 h (mL)	6075 (4550–6470)	5550 (4405–7900)	525 (−1833, 2883)
Urinary frequency (no. of micturitions) per 72 h	27 (25–32)	18 (16–19)	9 (5, 13)
Average voided volume per micturition (mL)	242 (161–261)	302 (236–419)	−60 (−192, 72)
Voided volume per 72 h (mL)	6285 (4805–7580)	5020 (4145–7163)	1265 (−924, 3454)
No. of urinary urgency episodes per 72 h	13 (9–18)	0 (0–0)	13 (7, 18)
No. of urgency urinary incontinence episodes per 72 h	2 (0–5)	0 (0–0)	2 (−1, 5)
**Questionnaires**			
ICIQ-FLUTS (score range 0–48)	13 (12–16)	2 (1–3)	11 (8, 13)
*Filling (score range 0–16)*	7 (5–8)	1 (1–2)	6 (4, 8)
*Voiding (score range 0–12)*	1 (0–3)	0 (0–1)	1 (−1, 3)
*Incontinence (score range 0–20)*	5 (4–7)	0 (0–1)	5 (3, 6)
OAB-q SF			
*Symptoms (transformed score range 0–100)*	52 (35–70)	3 (0–8)	48 (32, 65)
*Quality of life (transformed score range 0–100)*	65 (58–69)	98 (97–100)	−34 (−42, −25)
HADS			
*Anxiety (score range 0–21, cut-off ≤ 7)*	6 (4.5–8.5)	2.5 (1.5–4)	3.5 (1.2, 5.8)
*Depression (score range 0–21, cut-off ≤ 7)*	3.5 (2.5–4)	0 (0–0.5)	3.5 (2.2, 4.8)
MMSE *(score range 0–30, cut-off ≥ 24)*	29.5 (29–30)	29 (29–30)	0.5 (−0.7, 1.7)
**Urodynamic investigation**			
Filling cystometry			
*First sensation of filling (mL)*	80 (65–110)	23 (8–150)	58 (−34, 149)
*First desire to void (mL)*	163 (120–293)	268 (128–373)	−105 (−267, 57)
*Strong desire to void (mL)*	305 (190–420)	520 (388–660)	−215 (−413, −17)
*Maximum cystometric capacity (mL)*	378 (258–498)	643 (550–720)	−265 (−418, −112)
*Maximum detrusor pressure (cmH_2_O)*	10 (3–22)	3.5 (2.5–8)	7 (−7, 20)
*Detrusor overactivity, n (proportion)*	7 (0.58)	0 (0)	0.58 (0.3, 0.86)
Pressure-Flow-Study			
*Voided volume (mL)*	360 (155–390)	643 (550–723)	−283 (−413, −152)
*Maximum flow rate (mL/s)*	21 (11–33)	23 (21–33)	−2 (−15, 12)
*Maximum detrusor pressure (cmH_2_O)*	33 (17–74)	37 (26–42)	−4 (−30, 22)
*Detrusor pressure at maximum flow rate (cmH_2_O)*	25 (11–40)	26 (19–32)	−1 (−21, 19)
*Post void residual volume (mL)*	0 (0–45)	0 (0–0)	0 (−28, 28)

All continuous and discrete variables are presented as medians and range from lower (25th percentile) to upper (75th percentile) quartile, except for the dichotomous variable (presence of detrusor overactivity), which is presented as a number and proportion. Group differences were calculated by subtracting the median value of the healthy control population from the median value of the OAB group, and 95% confidence intervals (CIs) were obtained by bootstrapping (1000 iterations), except for the dichotomous variable (presence of detrusor overactivity) for which group difference and 95% confidence intervals were derived from a two-sample test of proportions. HADS (hospital anxiety and depression scale), HC (healthy control), ICIQ-FLUTS (international consultation on incontinence modular questionnaire on female lower urinary tract symptoms), MMSE (Mini-Mental State Examination), OAB (overactive bladder), OAB-q SF (overactive bladder questionnaire short form).

**Table 3:** ROI-to-ROI connectivity.

ROI-to-ROI	t-Value	Cohen’s d
rPFC L—OFC R	4.80	2.05
pSTG R—pSMG L	4.46	1.90
ACC—OFC R	4.31	1.84
PO L—OFC R	4.13	1.76
ICC L—OFC R	4.10	1.75
IFG L—SMG L	3.98	1.70
pSMG L—pMTG R	3.94	1.68
FO L—SMG L	3.92	1.67
SCC R—OFC R	3.91	1.67
SCC L—OFC R	3.85	1.64
pHG L—OFC R	−3.82	−1.63

List of all the ROI-to-ROI connections, as displayed in Figure 2A. This table additionally shows for each connection the t-values and Cohen’s d as measure of effect size. ACC (anterior cingulate cortex), FO L (frontal operculum, left), ICC L (intracalcarine sulcus, left), IFG L (inferior frontal gyrus, left), OFC R (orbitofrontal cortex, right), pHG L (parahippocampal gyrus, left), pMTG R (posterior middle temporal gyrus, right), PO L (parietal operculum, left), pSMG L (posterior supramarginal gyrus, right), pSTG R (posterior superior temporal gyrus, right), rPFC L (rostral prefrontal cortex, left), SCC R/L (supracalcarine cortex, right and left), SMG L (supramarginal gyrus, left).

**Table 4:** Seed-to-voxel connectivity.

Contrast	Seed	Connected Brain Region	Cluster Size	MNI—Coordinates			t-Value	Cohen’s d
				X	Y	Z		
OAB < HC	right AIC	ACC	77	0	8	28	5.24	2.23
		IFG	28	48	24	16	6.27	2.67
		SPL	23	20	−46	54	5.77	2.46
		MFG	20	40	0	46	4.59	1.96
		Calcarine cortex	19	14	−86	2	5.14	2.19
		Cerebellum	18	−24	−78	−26	4.82	2.06
	right VLPFC	SFG	63	16	8	62	5.40	2.30
OAB > HC	right AIC	No connectivity differences at the specified threshold						
	right VLPFC	No connectivity differences at the specified threshold						

List of all results from the seed to voxel analysis. Results are shown at p (voxel-threshold) < 0.001 with cluster level correction at a region of interest level (*p* < 0.05). ACC (anterior cingulate cortex), AIC (anterior insular cortex), HC (healthy control), IFG (inferior frontal gyrus), MFG (middle frontal gyrus), OAB (overactive bladder), SFG (superior frontal gyrus), SPL (superior parietal lobe), VLPFC (ventrolateral prefrontal cortex).

**Table 5:** (**a**) Seed-to-voxel connectivity in OAB patients in correlation to urinary urgency. (**b**) Seed-to-voxel connectivity in OAB patients in correlation to urinary frequency. (**c**) Seed-to-voxel connectivity in OAB patients in correlation to urgency urinary incontinence.

**(a)**														
**Correlation Direction**	**Seed: Right AIC**					**Seed: Right VLPFC**								
	**Brain Region**	**Cluster Size**	**MNI—Coordinates**			**Brain Region**		**Cluster Size**		**MNI—Coordinates**				
			**X**	**Y**	**Z**					**X**		**Y**		**Z**
positive	MTG	56	36	2	−26	THA		27		−14		−24		14
	MTG	42	−66	−22	−10									
	TP	35	−42	8	−40									
	OP	31	−28	−96	10									
	LOC	26	−40	−86	10									
negative	postCG	58	14	−42	78	No correlations at the specified threshold								
	postCG	41	−12	−42	80									
	preCG	33	−56	0	20									
	IFG	30	52	10	10									
**(b)**														
**Correlation Direction**	**Seed: Right AIC**					**Seed: Right VLPFC**								
	**Brain Region**	**Cluster Size**	**MNI—Coordinates**			**Brain Region**	**Cluster Size**		**MNI—Coordinates**					
			**X**	**Y**	**Z**				**X**		**Y**		**Z**	
positive	LOC	15	−56	−8	26	No correlations at the specified threshold								
negative	postCG	21	−22	48	−6									
	FP	11	30	−88	4									
**(c)**														
**Correlation Direction**	**Seed: Right AIC**					**Seed: Right VLPFC**								
	**Brain Region**	**Cluster Size**	**MNI—** **Coordinates**			**Brain Region**	**Cluster Size**		**MNI—** **Coordinates**					
			**X**	**Y**	**Z**				**X**		**Y**		**Z**	
positive	CERE	13	40	−62	−24	Brain stem	14		4		−12		−36	
negative	LOC	37	−16	−64	46	PUT	17		22		8		−4	
	PREC	13	−6	−46	56	LG	13		−28		−48		−6	
	SPL	12	46	−42	58	IFG	12		−46		14		16	

Correlation of seed-to-voxel connectivity to the number of urinary urgency episodes (a), urinary frequency (b), and urgency urinary incontinence episodes (c) per 72 h taken from the 3-day bladder diary. Results are shown at *p* (voxel-threshold) < 0.001 with cluster level correction at a region of interest level (*p* < 0.05). AIC (anterior insular cortex), CERE (cerebellum), FP (frontal pole), IFG (inferior frontal gyrus), LG (lingual gyrus), LOC (lateral occipital cortex), MTG (middle temporal gyrus), OP (occipital pole), postCG (postcentral gyrus), preCG (precentral gyrus), PREC (precuneus), PUT (putamen), SPL (superior parietal lobe), THA (Thalamus), TP (temporal pole), VLPFC (ventrolateral prefrontal cortex).

## Data Availability

The data presented in this study are available on request from the corresponding author.

## Supplementary Materials

The following supporting information can be downloaded at: https://www.mdpi.com/article/10.3390/biomedicines11061640/s1, Figure S1: Visualization of the results from the seed to voxel analysis using the periaqueductal gray (PAG) as seed region. References [79,80,81] are cited in Supplementary Materials.
