# Supplementary material for: Abnormal Resting-State Network Presence in Females with Overactive Bladder
Source: Biomedicines. 2023 Jun 5;11(6):1640. doi: 10.3390/biomedicines11061640 (PMC10296293; doi:10.3390/biomedicines11061640)
Supplement: Supplementary file 1 [file biomedicines-11-01640-s001.zip › biomedicines-2293093-supplementary.pdf]

Supplementary material

As the periaqueductal gray (PAG) has also been described in numerous neuroimaging studies investigating the (dys)function of the lower urinary tract in humans and, in relation to its anatomy and basic/general function, a relevant role in the supraspinal control of the lower urinary tract has also been attributed [1], we additionally perform a seed-to-voxel analysis with the PAG as seed region.
The OAB group demonstrated a stronger connectivity from the PAG to the right occipital lobe compared to HC whereas the HC group demonstrated stronger connectivity from the PAG to the right thalamus compared to the OAB group (Figure S1).
The functional connection between the PAG and the thalamus is important for modulating sensory information and regulating a range of physiological and emotional processes, including LUT control [2]. A reduced PAG - thalamus connectivity could provide further evidence for our hypothesis that OAB is associated with altered sensory processing and attentional control at the supraspinal level.
The connection of the PAG to the occipital lobe, which is primarily responsible for visual processing, in the context of LUT control is less clear or established. The connection of the PAG to the occipital pole has been described to be involved in crossmodal sensory processing in healthy adults, but has also been found activated during mind wandering away from pain [3]. The latter may be also a strategy relevant in OAB.

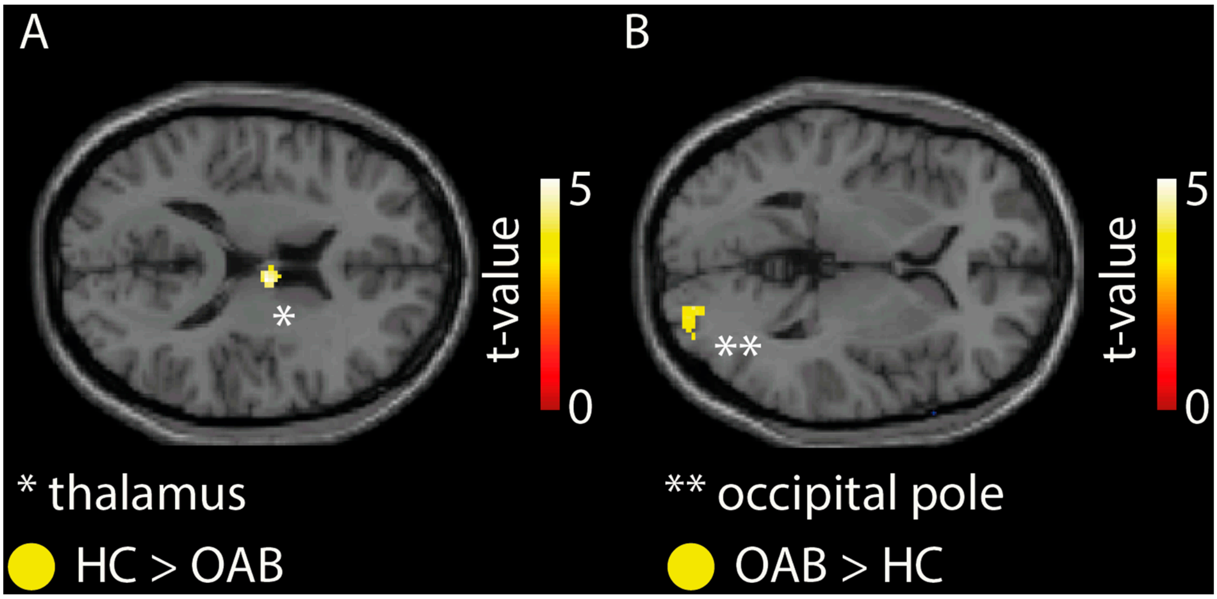

Figure S1. Visualization of the results from the seed to voxel analysis using the periaqueductal gray (PAG) as seed region. A) Overactive bladder (OAB) patients showed weaker connectivity compared to healthy controls (HC) from the seed to the right thalamus (t= 4.95, k = 41 voxels, Cohen's d = 2.1). B) OAB patients showed stronger connectivity compared to HC from the seed region to the right occipital pole (t = 4.73, k = 72 voxels, Cohen's d = 2.0). Results are shown at p (voxel-threshold) < 0.001 with cluster level correction at region of interest level (p < 0.05).

1. Fowler, C.J.; Griffiths, D.J. A decade of functional brain imaging applied to bladder control. *Neurourol Urodyn* **2010**, *29*, 49-55, doi:10.1002/nau.20740.
2. Zare, A.; Jahanshahi, A.; Rahnama'i, M.S.; Schipper, S.; van Koeveringe, G.A. The Role of the Periaqueductal Gray Matter in Lower Urinary Tract Function. *Mol Neurobiol* **2019**, *56*, 920-934, doi:10.1007/s12035-018-1131-8.
3. Coulombe, M.A.; Erpelding, N.; Kucyi, A.; Davis, K.D. Intrinsic functional connectivity of periaqueductal gray subregions in humans. *Hum Brain Mapp* **2016**, *37*, 1514-1530, doi:10.1002/hbm.23117.
